# Point-of-Care Diagnostics of COVID-19: From Current Work to Future Perspectives

**DOI:** 10.3390/s20154289

**Published:** 2020-07-31

**Authors:** Heba A. Hussein, Rabeay Y. A. Hassan, Marco Chino, Ferdinando Febbraio

**Affiliations:** 1 Virology Department, Animal Health Research Institute (AHRI), Agricultural Research Center (ARC), Dokki, Giza 12619, Egypt; hebaahmed@ahri.gov.eg; 2 Nanoscience Program, University of Science and Technology (UST), Zewail City of Science and Technology, 6th October City, Giza 12578, Egypt; ryounes@zewailcity.edu.eg; 3 Applied Organic Chemistry Department, National Research Centre (NRC), Dokki, Giza 12622, Egypt; 4 Department of Chemical Sciences, University of Naples “Federico II”. Via Cintia 21, 80126 Napoli, Italy; marco.chino@unina.it; 5 Institute of Biochemistry and Cell Biology, National Research Council (CNR), Via P. Castellino 111, 80131 Naples, Italy

**Keywords:** biosensors, SARS-CoV-2, COVID-19, point-of-care devices, immunoassays, spike protein

## Abstract

Coronaviruses have received global concern since 2003, when an outbreak caused by SARS-CoV emerged in China. Later on, in 2012, the Middle-East respiratory syndrome spread in Saudi Arabia, caused by MERS-CoV. Currently, the global crisis is caused by the pandemic SARS-CoV-2, which belongs to the same lineage of SARS-CoV. In response to the urgent need of diagnostic tools, several lab-based and biosensing techniques have been proposed so far. Five main areas have been individuated and discussed in terms of their strengths and weaknesses. The cell-culture detection and the microneutralization tests are still considered highly reliable methods. The genetic screening, featuring the well-established Real-time polymerase chain reaction (RT-PCR), represents the gold standard for virus detection in nasopharyngeal swabs. On the other side, immunoassays were developed, either by screening/antigen recognition of IgM/IgG or by detecting the whole virus, in blood and sera. Next, proteomic mass-spectrometry (MS)-based methodologies have also been proposed for the analysis of swab samples. Finally, virus-biosensing devices were efficiently designed. Both electrochemical immunosensors and eye-based technologies have been described, showing detection times lower than 10 min after swab introduction. Alternative to swab-based techniques, lateral flow point-of-care immunoassays are already commercially available for the analysis of blood samples. Such biosensing devices hold the advantage of being portable for on-site testing in hospitals, airports, and hotspots, virtually without any sample treatment or complicated lab precautions.

## 1. Introduction

### 1.1. The Virus Origin and History

**Figure 1:**
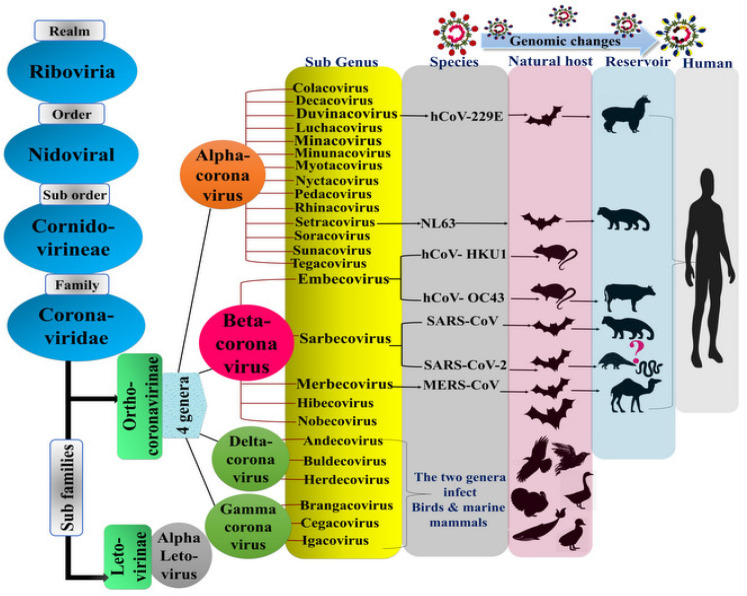
Coronavirus taxonomy according to the International Committee on Taxonomy of Viruses (ICTV) showing the classification of the SARS-CoV-2. Genomic changes of the virus enabled reemergence and jumping from the original host (bats) to the intermediate host, and finally to humans, in a tortuous evolutive pattern.

The taxonomy of coronaviruses (CoVs) has been recently classified, according to the International Committee on Taxonomy of Viruses (ICTV), to order *Nidovirales*, family *Coronavirideae*. Of the two subfamilies, *Orthocoronavirinae* was classified into four genera, based on the serological relationship and the sequence identity of the replicase regions: *alpha*, *beta*, *delta*, and *gammacoronaviruses* [1] (Figure 1). Each genus is able to infect a wide variety of host species. *Alpha* and *beta* genera, representing the ⅔ of known coronaviruses, are the most studied because they target mammals, including humans [2], whereas *gamma* and *delta* infect birds and in one case *Cetacea*. Several subgenera may be attributed to CoVs according to this classification. In particular, genus *alphacoronavirus* contains 14 subgenera, with 19 viral species. *Betacoronavirus* genus contains 5 subgenera and 14 viral species on its side. *Gamma* and *deltacoronaviruses* include three subgenera each, with seven and five species, respectively [2] (Figure 1).

The importance of the betacoronaviruses (β-CoVs) emerged from their ability to genetically evolve inside the host body and then in the intermediate putative host, which is, in turn, the suitable media to jump towards humans. Bats are the natural reservoir of most CoVs except OC43 and HKU1, which originated from rodents (*embecovirus*) [3]. Since 1960, 30% of respiratory illnesses were caused by the pneumotropic coronaviruses, including human coronaviruses (hCoVs) 229E, OC43, NL63, and HKU1, and were deemed to be nonfatal, until 2002, when the shocking global outbreak of severe acute respiratory syndrome (SARS) appeared in Guangdong province, China [4]. SARS was associated with high mortality rates and subsequently disseminated through other surrounding countries, including Thailand, Singapore, Vietnam, Hong Kong, Taiwan, and extended to the United States of America. Afterwards, the World Health Organization (WHO) and the Centers for Disease Control and Prevention (CDC) declared a global state of emergency, caused by the SARS-CoV [4,5,6]. 

A bat virus from a different subgenus again disclosed its presence in June 2012, especially in Saudi Arabia and generally in the Gulf region, and then extended towards many countries in Asia, Africa, Europe, and America. This disease is also known as the Middle East Respiratory Syndrome Coronavirus (MERS-CoV) [5,6,7]. The MERS-CoV that was firstly isolated and identified by Dr. Ali M. Zaki, an Egyptian virologist working at a hospital in Kingdom of Saudi Arabia (KSA), was the only *betacoronavirus* belonging to *merbecovirus* infecting humans [5,8]. Despite their bat origins, SARS-CoV and MERS-CoV have been demonstrated to infect humans via an intermediate host, rather than through a direct infection from bats [9,10].

In this regard, serological studies showed the presence of cross-reactive antibodies against MERS-CoV in dromedary camels of many countries, including Oman, the Canary Islands, and Egypt. More recent studies of one specific patient who died in Jeddah (Kingdom of Saudi Arabia) in which dromedary to human close contact could be followed, suggested that direct cross-species transmission indeed happened [11]. Real-time polimerase chain reaction (RT-PCR) data showed 99.8% identity between the two isolated viruses from different species. Nevertheless, viruses are rapidly changing in their genome when interacting with hosts, and it is actually very difficult to find the same intact RNA in two different species. 

The other bat viruses, such as *Tylonycteris bat* coronavirus HKU4 (BtCoV-HKU4), *Pipistrellus bat* coronavirus HKU5 (BtCoV-HKU5) and *Rousettus bat* coronavirus HKU9 (BtCoV-HKU9), are not associated with human disease and belong to *hibeco* and *nobecovirus* subgenera [12].

Right now, the devastating outbreaks taking place around the globe are caused by severe acute respiratory syndrome Coronavirus-2 (SARS-CoV-2). The reemergence of a SARS disease was initially disclosed in Wuhan, China, in December 2019, and in March 2020, the World Health Organization announced the disease as a pandemic Coronavirus disease 2019 (COVID-19) [13]. The receptor protein sequencing and restricted genetic analyses showed similarities to many species, which have been referred to intermediate hosts, including pangolins, turtles, and snakes [14,15,16]. Recently, the genomic analysis of the CoVs isolated from the Malayan Pangolin showed high similarities to SARS-CoV-2 (counting 100%, 98.6%, 97.8%, and 90.7% sequence identities of E, M, N, and S genes, respectively) [17]. In addition, the receptor-binding domain (RBD) of the spike protein (*vide infra*) has virtually shown no differences from the one found in SARS-CoV-2 [17]. Furthermore, multiple sequence alignment showed close similarity between the SARS-CoV-2 genome and two bat viruses, BtSL-CoV-ZC45 (87.99% identity) and BtSL-CoV-ZXC21 (87.23% identity). Surprisingly, despite striking similarities among the novel SARS-CoV-2 and SARS-CoV, the former has quickly spread to the human population. This seems to be explained by the structural differences in the spike protein (*vide infra*) (76–78% sequence identity with SARS-CoV) among the coronaviruses [18,19]. 

The route of the transmission plays an important role in the pathogenesis and the severity of the virus infection. Such respiratory disease viruses disseminate among humans through the respiratory pathways, especially by airborne infectious aerosols and droplets. SARS-CoV-2 is mainly transmitted by direct contact with the infected persons via droplet and aerosol infection [20], in contrast with SARS-CoV and MERS-CoV for which limited transmission occurs, in particular through the nosocomial route [21,22].

### 1.2. General Characteristics of the SARS-CoV-2

**Figure 2:**
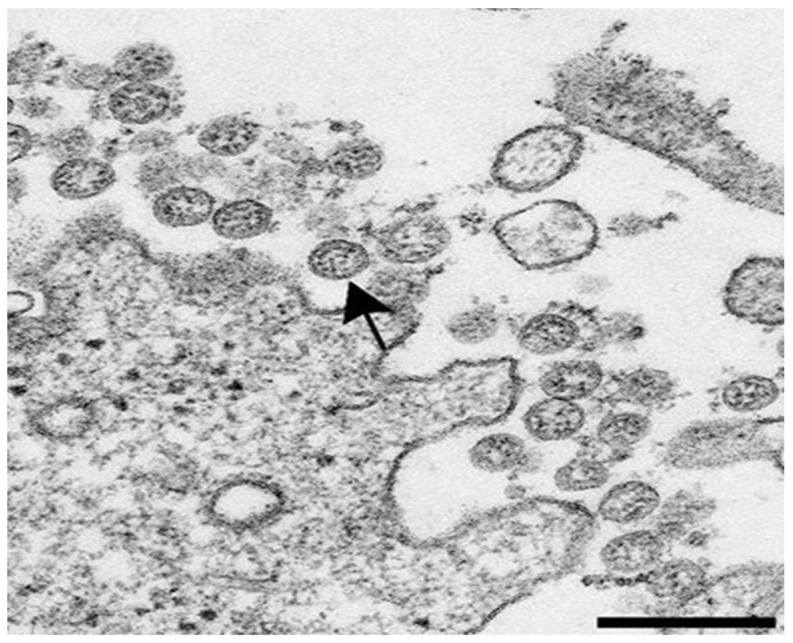
Transmission electron microscopy image of the SARS-CoV-2 isolated from patients showing extracellular spherical particles with cross-sections through the nucleocapsids (black dots). Arrow indicates a coronavirus virion budding from a cell. Scale bar indicates 200 nm. Published in [16] https://dx.doi.org/10.3201/eid2606.200516.

Coronaviruses are nonsegmented, positive-sense, single-stranded, and enveloped RNA viruses, sized between 65 and 145 nm. They have stalk-like projections ending with a peplomeric structure called the spike protein (S protein), which grants the coronaviruses their typical crown-like morphology (Figure 2). 

Coronaviruses are the most recombinogenic and mutative known viruses, at the moment [3]. Thus, the genetic recombination of some genomic subregions, associated with different independent origins inside the animal reservoirs, supports the circulation of the viral infection, recombination, and coinfection inside the reservoir and intermediate hosts.

**Figure 3:**
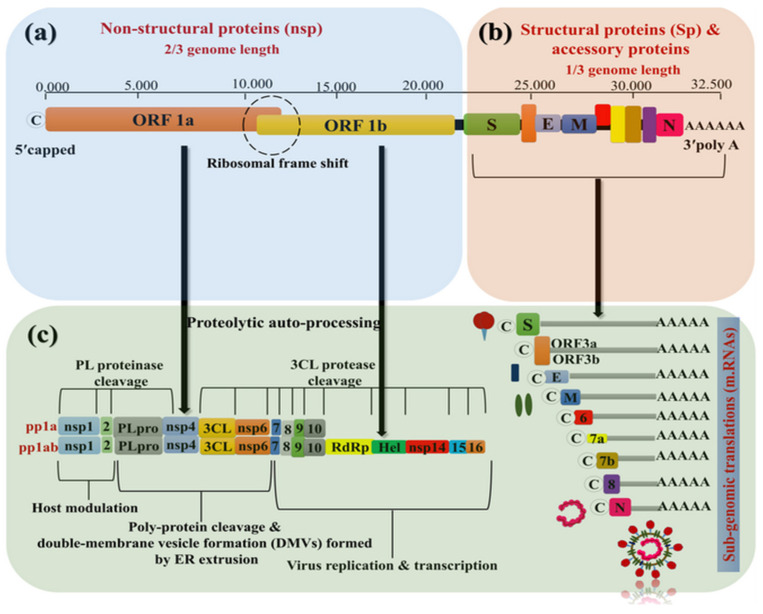
SARS-CoV-2 genetic map. (**a**) The genetic arrangement of the virus codons from 5’ capped to (**b**) 3’ poly-A tail, and (**c**) the post-translation process of the nonstructural, accessory, and structural proteins.

MERS-CoV and SARS-CoV have the largest genome, encompassing 27.9 and 30.1 kb, respectively [10,23]. The genomic mapping of the recent SARS-CoV-2 has been identified as 29.9 kb with 38% of G/C pairs [24]. The genomic strip stretched from a 5′ capped to a 3′ polyadenylated ending sequence, acting as an mRNA ready for the polyprotein translation by replicase enzyme. The subgenome contains between 6 and 11 open reading frames (ORFs). The first one (ORF1a/b) occupies two-thirds of the viral genome length encoding 16 nonstructural proteins (nsps) (Figure 3a), which assembled to form a membrane-associated viral replicase–transcriptase complex (RTC) [25,26]. 

These proteins are cleaved and activated by the viral enhanced chymotrypsin-like protease (3CL^pro^), protease (M^pro^), and one or two papains [27] (Figure 3c). The two polypeptides pp1a and pp1ab are produced due to the frame shifting between ORF1a and b by -1. The other part of the virus ORFs near the 3′ terminus (Figure 3b) encoded the viral structural proteins (sps), including spike (S), membrane (M), envelope (E), and nucleocapsid (N) proteins [28,29]. Furthermore, other accessory proteins are encoded in this part (Figure 3b,c) [29]. They are thought to interfere with the host’s innate immunity and to play a role in the virus replication process.

### 1.3. Cell Entry and the Role of the S Protein in the Virus Infectivity and Replication

**Figure 4:**
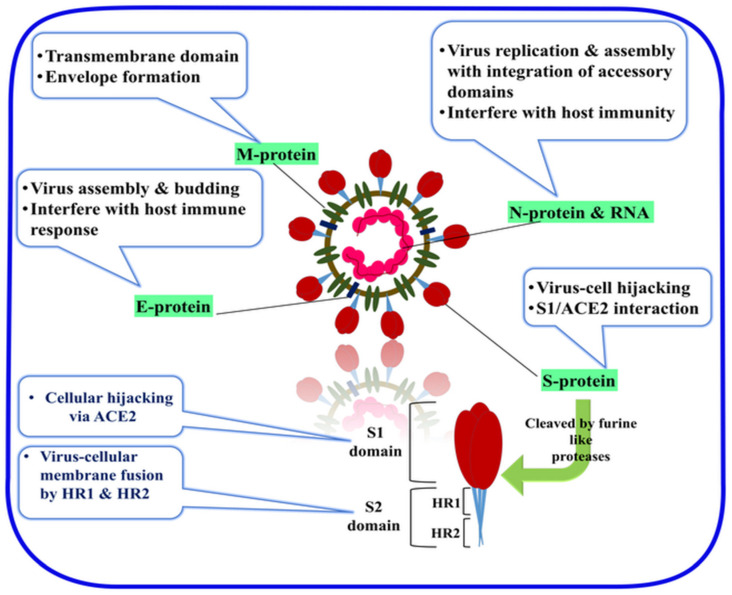
SARS-CoV-2 structural proteins, including membrane (M), envelope (E), nuclear (N), and spike (S) proteins. S protein cleaved by the host proteases into S1 and S2 domains.

β-CoVs encode a distinctive surface structure characterized by the S protein (~140 kDa), that has about 1104 to 1273 amino acids. This comprised the heavily N-linked glycosylated (~25 kDa N-linked glycans per monomer) homotrimers and was classified as a class I fusion protein. It is cleaved by the host cell furin-like proteases into two separate S1 and S2 domains, as shown in Figure 4 [30,31,32,33].

The virus hijacks the host–cell receptors via its RBD from the S1 portion. Meanwhile, the host proteases the cleaved domain S2, which is responsible for the membrane fusion, and forms the stalk of the spike protein [34]. The virus utilizes the host–cell determinants, the extracellular claw-like N-terminus peptidase domain (PD), and the C-terminal collectrin-like domain (CLD), which are cleaved by the angiotensin-converting enzyme 2 (ACE2), in the case of SARS-CoV and SARS-CoV-2. MERS-CoV instead utilizes the dipeptidyl peptidase-4 (DPP4) (CD26) receptor for the cellular invasion [34,35,36].

It is worth noticing that, the cellular integrin ACE2 belongs to the type I cell membrane protein receptors, which are expressed in most human tissues, including heart, colon, smooth intestine, skin, lymph nodes, liver, bile ducts, and brain, as well as in arterial and venous endothelial cells, arterial smooth-muscle cells in lungs, and kidney parietal epithelial cells. In addition, its expression on the surface of the alveolar epithelium of the lung and in the enterocytes of the small intestine gives the complete clue for the multitissue tropism of the SARS-CoV-2. The nasal epithelial and the upper respiratory tissues were, therefore, the primary selective site for the SARS-CoV-2 infection [37,38,39,40]. An independent folding of the RBD region from the remaining S protein, depending on the structural asset of the host, was also reported [41].

The cryogenic electron microscopy (cryo-EM) structure of the SARS-CoV showed that a conformational change of the trimeric RBD, accompanied by the partial rotation of one domain, was necessary to enable the receptor binding. Yan et al. [42] recently demonstrated by high-resolution cryo-EM that the structural alignment of the ACE2-RBD-B^0^AT1 ternary complex docked with the S protein of the SARS-CoV-2. The two S-protein trimers simultaneously bind to the ACE2 homodimer, and its high-affinity binding is supposed to facilitate the development of trap ligands and moieties, neutralizing antibodies for the inhibition of the viral infection [42].

The particularly critical residue Gln493 in RBD from SARS-CoV-2 provides the high affinity to human cell attachment, as well as with the other residue Asn501, which is compatible with cell binding but to a lesser extent [43]. Furthermore, the S protein from SARS-CoV-2 utilizes the ACE2 for the cellular entry, employing the cellular transmembrane protease serine 2 (TMPRSS2), for the S-cleavage and priming [44,45]. The TMPRSS2 is used as a host cell factor, which is critical for the spread of most viruses, including influenza A viruses and other coronaviruses [46,47]. In fact, it has been reported that the use of serine proteases or TMPRSS2 inhibitors blocks the virus’ entry into the lung and in vitro cells [48].

In other structural viral proteins, the membrane (M) protein (~25–30 kDa) is the most abundant structural protein in the virus, constituting the virus shape [49]. Furthermore, it is formed by three transmembrane domains and consists of a small N-terminal glycosylated ectodomain and C-terminal endo-domain (6–8 nm) into the viral particle [49]. The M protein folds as a dimer in the virus with two conformational changes that promote the membrane curvature to bind to the nucleocapsid [50]. The envelope (E) protein (~8–12 kDa) has a highly divergent distribution that consists of an N terminal ectodomain and C-terminal endo-domain with ion exchange activity [51]. The E protein facilitates the virus’ assembly and release—it is also required for virus pathogenesis [52]. The nucleocapsid (N) protein contributed to the RNA binding in a beads-on-a-string-type conformation via the phosphorylation of the N protein over the nonviral RNA [53,54] (Figure 4). The identified RNA substrates for the N protein include the transcriptional regulatory sequences (TRSs) and the genomic packaging signal. The genomic packaging signal binds to the C-terminal RNA binding domain, while the N protein binds to nsp3, the masterpiece of the replicase complex, and the M protein [55]. The interactions between these proteins provide the tethering of the virus RNA to the replicase–transcriptase complex (RTC) and afterward, the viral genome is packed into the virus capsule [54,55]. In some subsets of the hCoVs, including MERS-CoV, the hemagglutinin-esterase, by its acetyl-esterase activity, can bind to sialic acid on the surface of glycoproteins and enhance S-protein-mediated cell entry and infectivity through the mucosa [56,57,58] (Figure 3c).

## 2. Diagnosis of COVID-19

Many diagnostic methods were developed for the detection of hCoVs and/or the correlated diseases, including antigen detection, molecular identification, or antibody assessment by different immunoassays. Since hCoVs’ first emergence, the diagnostic assays were originally developed to meet the global needs on the commercial level. Sophisticated research trials for such assays have been performed over the last twenty years. Unfortunately, the ongoing global crisis represented a highly demanding benchmark for sensitivity and selectivity of the methods adopted to detect SARS-CoV-2. In this article, previously developed diagnostic methods for the detection of SARS-CoV and MERS-CoV will be discussed, as well as the newly developed diagnostic assays.

### 2.1. Laboratory-Based Cell Culture Detection

In the hypothesis that a suitable cell line for virus isolation and amplification is available, cytopathogenic effect (CPE) (e.g., cell rounding, agglomeration) and further evaluation of the tissue culture infective dose 50 (TCID_50_) can be used for virus titers determination. Nevertheless, a confirmatory testing for the identification of the specific virus type is required either by using genetic analysis (vide infra) or by microneutralization assay (MNA). The latter depends on the neutralization of the virus by specific antibodies when applied on the culture cells. However, neutralizing antibodies (nAbs) are not always available, either coming from purified antibodies or standard positive serum samples. MNA is useful in the determination of nAbs titers in sera from positive patients anyway. 

Further limitation for this typology of assays is represented by a high risk of virus propagation. Depending on the virus propagation on the cell culture system, the assay must be performed in biosafety level 3 (BSL3) laboratory due to the virus containment issues. Still, the microneutralization assay was the golden standard test for MERS-CoV detection [16,59,60]. As a matter of fact, both SARS-CoV and MERS-CoV can be cultivated on the Vero E6 cell line with a remarkable and measurable CPE. The CPE of the virus is observed 2–3 days post inoculation, for MERS-CoV [59]. The same time is, therefore, needed to check for positivity in human sera and, subsequently, the highest serum dilutions that completely protect the cells from CPE in half or all wells (MN_50_ and MN_100_, respectively). 

Lester et al. proposed an alternative method that could be used under BSL-2 conditions. They developed a microneutralization test in which S-protein-bearing vesicular stomatitis virus (VSV) pseudotype particles (VSV-MERS-CoV-S) were used in place of the highly dangerous MERS-CoV. Remarkably, the luciferase reporter gene was replacing the VSV glycoprotein G gene. In this way, when Vero cells were cultured in the presence of VSV-MERS-CoV-S and human serum positive to MERS-CoV neutralizing antibodies, luciferase expression could be assessed after 48 h of incubation. Chemiluminescence was found proportional to neutralizing antibody for a wide range of concentrations (40–1280 antibody titers), and the test was validated against human serum (52 samples). High sensitivity (five sera with neutralization titers below the limit of detection (LOD) of standard microneutralization were detectable by the VSV-MERS-CoV-S assay) and high specificity (as assessed against five human sera positive to five different hCoVs) were found [60].

Based on the previous experiences on SARS-CoV and MERS-CoV, SARS-CoV-2 has been cultivated on Vero E6 cells, as well as on TMPRSS2-expressed Vero E6 cell line, which were maintained in Dulbecco minimum essential medium (DMEM) and supplemented with 5% or 10% heat-inactivated fetal calf serum [16,61]. More recently, an MNA against SARS-CoV-2 has been developed, which can also be used for screening of anti-SARS-CoV-2 compounds in vitro [62]. Such an assay is extremely useful for this kind of research, and it represents an invaluable tool in studying the virus biology. However, virus isolation needs 2 days and MNA at least 4 days, making it practically and commercially unaffordable both in diagnosis and monitoring of the virus spread in the population, especially if we consider the strict limitation in terms of biosafety (BSL3 for SARS-CoV-2).

### 2.2. Laboratory-Based Gene Detection

The virus propagation needs a BSL3 laboratory, therefore current hCoVs diagnosis was carried out by the reverse-transcriptase polymerase chain reaction (RT-PCR). The point-of-care multiplex RT-PCR is implemented in laboratories using clinical infected samples (nasopharyngeal and nasal swabs). However, samples from patients were subjected to RNA extraction, inactivating the virus and allowing analysis in hospital laboratories. The results are positive for genes encoding the internal RNA-dependent RNA polymerase and the S protein of these viruses [63].

**Figure 5:**
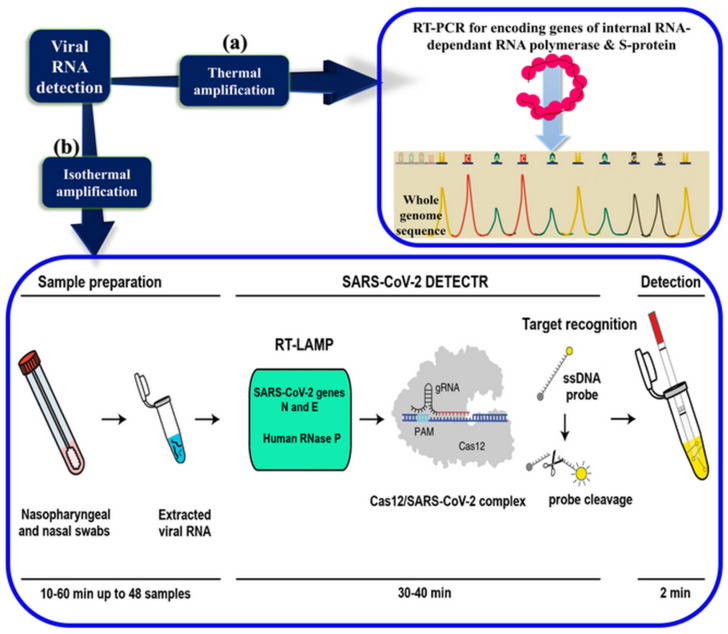
COVID-19 molecular diagnostic methods. (**a**) Virus molecular characterization by RT-PCR and whole-genome sequencing, (**b**) development of the CRISPR-Cas12 in the virus detection via the isothermal amplification of the viral genome.

Subsequently, for confutation, the whole genome sequencing should be performed by a sequence-independent single-primer amplification approach, e.g., using the Oxford Nanopore MinION device, which is supplemented by Sanger sequencing (Figure 5a). The similarities between the several lineages is identified by the phylogenetic analysis based on the data provided from GenBank [64]. However, the accuracy and sensitivity of RT-PCR relies on many technical factors, including the sampling and handling procedures and the quality of RNA extraction [65]. Thus, the ongoing current situation caused by the SARS-CoV-2 forces scientists around the globe to develop new diagnostic methods.

Recently, a 40-min CRISPR-Cas12-based lateral flow assay has been developed, called SARS-CoV-2 DNA Endonuclease-Targeted CRISPR Trans Reporter (DETECTR) for the detection of SARS-CoV-2 in the oropharyngeal and the nasopharyngeal swabs from the infected persons after the RNA extraction (Figure 5b). The assay utilizes the simultaneous reverse transcription in the isothermal amplification using the loop-mediated amplification technique (RT-LAMP), followed by Cas12 detection of the amplified virus sequence. After the reporter molecule cleavage confirms the virus detection, a lateral flow strip is used to confirm the presence or absence of a target virus. This diagnostic method provides a faster and trustable (95% and 100% for positive and negative predictive agreement, respectively) alternative to the SARS-CoV-2 RT-PCR assay performed in the US Centers for Disease Control and Prevention [66]. Although DETECTR is still a lab-based technique, it could represent a future gold standard for SARS-CoV-2 gene detection. Moreover, the Cas12 system is already commercially available, but an upgrade of laboratory equipment is still required to perform the assay. 

### 2.3. Laboratory-Based Immunological Detection

The serological immunoassays for CoVs were developed to enable high-throughput laboratory tools alternative to the RT-PCR based technique, which is time-consuming and requires pretreatment of samples for RNA extraction. However, in practice, such assays are dependent by the limit of detection of Ab titers, as well as the time of actual immunological response, which can start as early as 4 days after the onset of illness [67]. Therefore, they have been adopted as further support to RT-PCR in order to reach a higher positive detection rate [68]. Moreover, given that IgG concentration in serum may be high enough even after a few months [69], such assays are very useful in determining virus spread in the population and outbreak monitoring.

Severance et al. developed an enzyme-linked immunosorbent assay based on the coating with the tagged amino- and carboxy-terminal recombinant nucleocapsid antigens of the hCoV-229E, HKU1, NL63, and OC43, which were previously cloned into baculovirus and expressed in the insect cells [62]. The sero-surveillance of IgG in the human sera of 196 adults against each virus type indicated a high exposure rate to OC43, 229E, and NL63 of 90.8%, 91.3%, and 91.8%, respectively, and a moderate one to HKU1 (59.2%) among individuals in this population. In addition, the seropositivity and antibody levels appeared significantly associated with smoking (OC43) and socioeconomic (NL63) status. Furthermore, a high-level of immunoreactivity for each of the tested hCoVs was significantly associated with summer season [70].

Trivedi et al. carried out separate screening tests of IgG in human serum after covalent conjugation of purified His-tagged *rec*N (whole or truncated) of the six hCoVs 229E, NL63, OC43, HKU1, SARS-CoV, and MERS-CoV on multiplex magnetic microsphere immunoassay (MMIA), in *rec*N monoplex and *rec*N multiplex immunoassay sets. The tests were performed on paired sera samples with no cross-reactivity between mono and multiplex settings, obtaining a sensitivity and specificity of 86% and 84%, respectively [71]. 

There are many diagnostic methods based on the S protein for the MERS-CoV because of its role in eliciting neutralizing antibodies from the patients [72]. Some of these tests are used to track the spread of viruses among species (intermediate and reservoir hosts). As previously discussed, the MERS-CoV originated from the *merbecovirus* hCoVs in bats. The detection of the virus in a wide range of animal species, such as dromedary camels, facilitates tracking of the virus. In addition, it helped in identifying the ancestor of the MERS-CoV, which is also a possible route to identify monoclonal antibodies against an N protein [11]. In turn, such antibodies can be repurposed for the diagnosis of the MERS-CoV in the human sera. Therefore, such assays would also be used for the detection of *merbecovirus* β-CoVs in general, which originated from bats and still remain intraspecies, including bat-CoVs HKU4 and HKU5. This assay is considered a rapid on-site screening and tracking test for detection of *merbecovirus* hCoVs [73]. The commercialized semiquantitative enzyme-linked immunosorbent assay (ELISA) (EUROIMMUN, Lübeck, Germany) for MERS-CoV specific IgG antibodies in camel sera and plasma uses plates coated by purified S1 antigen of MERS-CoV [74]. However, for virus diagnosis, the ELISA assay needs to be confirmed by other standard methods, such as RT-PCR [65] or a pseudo-particle microneutralization test [60].

Despite the references about previous hCoVs, the RT-PCR still is the golden standard diagnostic method for detection and screening of the SARS-CoV-2. However, this tool takes a time ranging 3 to 5 h in sample preparation for the genome extraction. Still, an extremely high (98.6%) detection rate is observed when RT-PCR is performed in conjunction with IgM/IgG ELISA as compared to the genetic analysis alone (51.9%) [68].

In parallel, serological surveillance against SARS-CoV-2 mainly relies on screening of viruses through immunological responses of infected patients who produce IgM and IgG as a defensive immunological shield against the virus immunogenic proteins. As reported by Hou et al., antibodies against SARS-CoV-2 could be already revealed after 6 days from symptoms onset [69]. The IgM is detected in early stage disease about 2 days post infection, while the IgG is detected at a late stage (8 days of infection). Being the IgM and the IgG against SARS-CoV-2 indicative of a recent and prolonged viral infection, respectively, it is preferable to design an assay able to detect both immunoglobulins in a way to get detailed information on the ongoing disease. 

**Figure 6:**
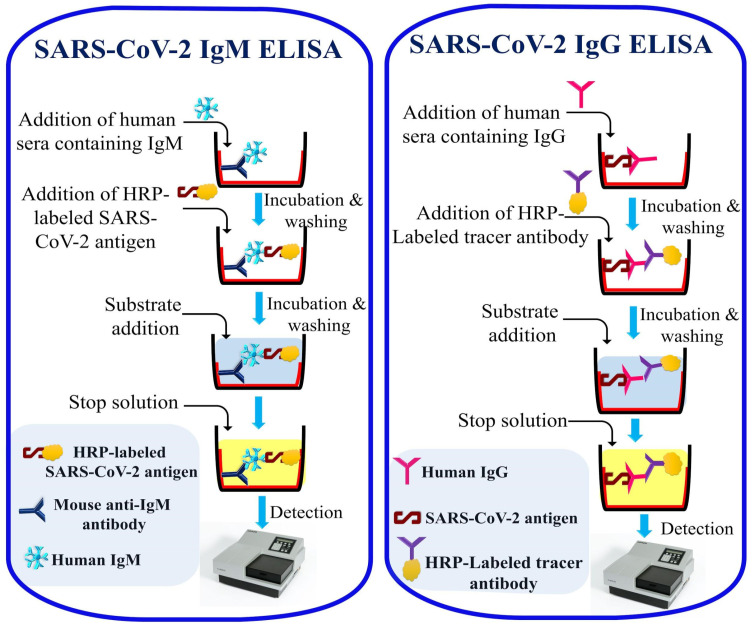
SARS-CoV-2 IgM/IgG dependent enzyme-linked immunoassay (ELISA), (**a**) SARS-CoV-2 IgM ELISA; (**b**) SARS-CoV-2 IgG ELISA.

Serological laboratory assays have been tested for the screening of IgG and IgM against SARS-CoV-2, based on the classical ELISA test (Figure 6) [75,76]. The ELISA test for human IgM uses microplates coated with mouse antihuman IgM monoclonal antibodies. After the formation of the human-IgM/mouse-anti-IgM complex, the detection depends on the addition of the HRP-labeled SARS-CoV-2 antigen that binds to specific human antibodies (Figure 6a). Whereas, the indirect IgG detection by ELISA uses plates coated by the recombinant antigen of the S protein of the SARS-CoV-2. The HRP-conjugated monoclonal mouse antihuman IgG recognizes the anti-S-protein-IgG present in the serum samples of patients (Figure 6b) [75]. 

**Figure 7:**
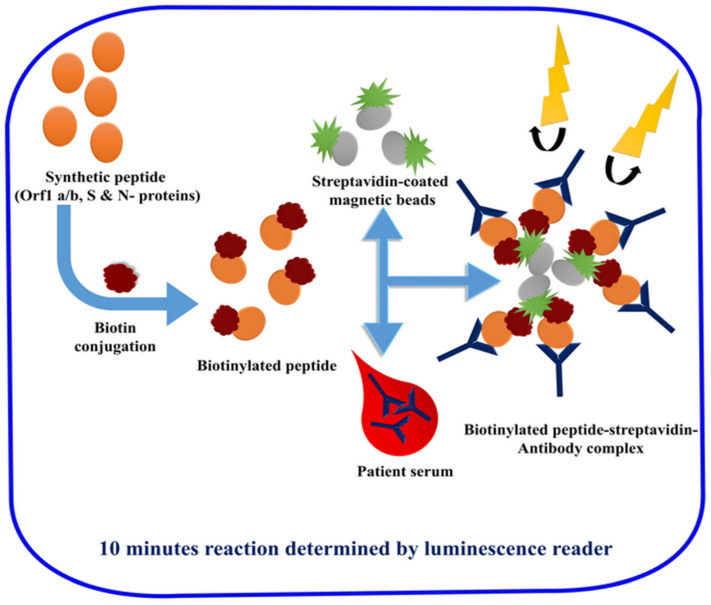
Peptide-dependent magnetic chemiluminescence enzyme immunoassay.

In addition, a luminescent immunoassay has been developed by using twenty synthetic peptide antigens identified by a genomic analysis in GenBank (NC-045512.1) of the ORF1a/b, S protein, and N protein regions (Figure 7) [77]. 

Peptides were labeled with biotin, and these biotinylated peptides were bound to the streptavidin-coated magnetic beads. Sera were then mixed with the conjugated beads, and after exhaustive washing cycles by magnetic precipitation, the luminescence detection was performed by the addition of chemiluminescent reagent. Only one peptide from S protein, which is giving the best result, was selected. This developed luminescent immunoassay was used as a tool for the detection of anti-SARS-CoV-2 antibodies (IgM and IgG, after separate assays) in the infected sera samples. The resulting data was analyzed by calculating the best cut-off value helping in the discrimination between healthy and the RT-PCR confirmed positive patients. When the signal/cut-off (S/C) ratio was higher than 1, the sample is treated as positive. However, the selectivity is not very high (71.4% for IgG and 57.2% for IgM). A combination of the two antibodies enhanced the detection rate to a final 81.5% (225 of 276) [77]. In this regard, the suitability of such techniques in being a valuable alternative to PCR-based diagnosis is highly limited, and positive sera should be confirmed using the RT-PCR anyway. Nevertheless, given the generally high sensitivity of chemiluminescence-based assays [78], there should be ample room to improve sensitivity and specificity (e.g., by adopting better-designed epitopes). 

### 2.4. Laboratory-Based Omic Detection

As COVID-19 becomes a pandemic, in concomitance with the genetic and serological assays, an emerging contribution by omics methodologies for the diagnosis and monitoring of SARS-CoV-2 infection has been observed. A collaboration among researchers working in mass spectrometry (MS) laboratories around the world, called the COVID-19 MS Coalition [79], represents an effort to share through open datasets molecular and structural information, as well as methodology (sample collection protocols, data generation, etc.) on SARS-CoV-2 in humans.

The considerable genetic and structural information allowed scientists to develop new MS-based analysis and to obtain rapid, precise, and reproducible diagnostic information that could complement the current diagnostic techniques [80]. MS-based methods for the detection in patient samples of the viral nucleocapsid N protein, the most abundant protein of the SARS-CoV-2, have been developed. Bezstarosti et al. demonstrated the potential of targeted MS proteomic technologies in identifying N protein with a LOD in the mid-attomole range (approximately 10,000 SARS-CoV-2 particles) in samples of virus-infected Vero cells, using Orbitrap Eclipse mass spectrometer [81]. Ihling et al. described an MS method, specifically identifying a unique peptide set originating from tryptic digestion of SARS-CoV-2 nucleoprotein from gargle solution samples of COVID-19 patients [82]. Similarly, Nikolaev et al. described the detection by LC-MS/MS of a unique set of tryptic peptides, produced from the proteolysis of the N protein of SARS-CoV-2 virus, in nasopharynx epithelial swabs collected from patients with CODIV-19 diagnosticated by RT-qPCR [83]. The main protocol provides the physico-chemical inactivation of the virus by heating and the addition of isopropanol, followed by sample pretreatment (lyophilization, alkylation, centrifugation, etc.) and tryptic digestion of the proteins from the swabs. Finally, a low-cost high-throughput MS platform for COVID-19 clinical diagnosis was described by Messner et al. [84]. Authors introduced a new pipeline for the samples’ preparation, using short-gradient high-flow LC-MS, in order to meet clinical implementation and increase the sample throughput and quantification. They report 27 biomarkers that distinguish mild and severe forms of COVID-19, some of which have potential as therapeutic targets.

Although these novel applications seem to mark the transformation of proteomics from a research tool into a diagnostic tool for clinical use, these studies are still preliminary, because all the articles describing such approaches are from preprint databases and still not peer reviewed. Unfortunately, they cannot be considered as conclusive or as established information. SARS-CoV-2 detection in patient biological samples, such as swabs or body fluids, using omics technologies largely depends on the amount of viral proteins present in such samples, so further research is required to demonstrate the sufficient sensitivity and precision of these tools. In fact, the use of LC-MS based techniques for pathogen detection, in particular viruses, is still strictly limited to research laboratories and only in a few cases could be used for diagnosis of patients. Other difficulties can be generally related to the time required for sample pretreatment and analysis, the high cost of instruments, together with the necessary high skill of personnel.

### 2.5. Point-of-Care and Standalone Biosensing Devices

**Figure 8:**
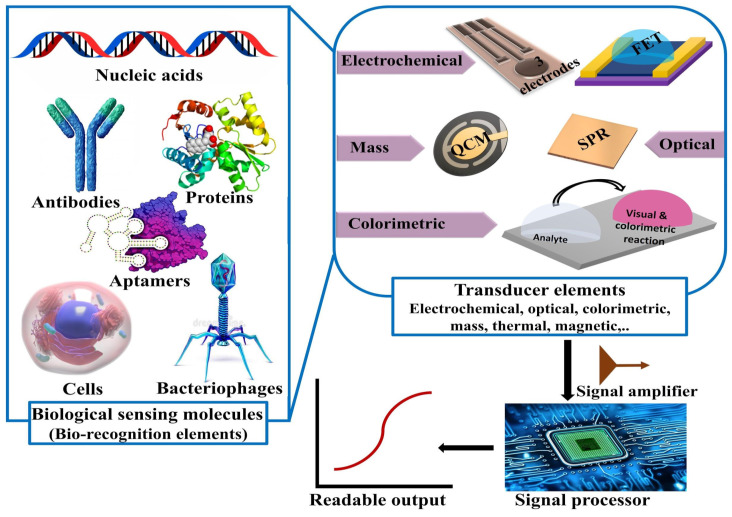
Main components of biosensors.

Biosensors are devices used to detect the presence or concentration of a biological analyte, such as a biomolecule, a biological structure, or a microorganism. A fully functional biosensor is generally composed of a recognition element that detects a certain molecular component(s) in the sample under investigation. Next, the recognition event is detected via the deployment of different transducers (e.g., electrochemical, optical, colorimetric, or mass change), which capture various signals to be further amplified and processed for data analysis. The advantages of the biosensing techniques are the cost-effectiveness, small sample requirement, reproducibility, fast detection, extreme sensitivity, ability to miniaturize, and user convenience. A brief description about the main components of a general biosensor is depicted in Figure 8.

It is worth noticing that the biosensing devices are useful in clinical detection, in which they provide (i) sensitive, selective, and immediate measurement of small amounts of analyte, (ii) robust and easy to perform procedure, (iii) the possibility for on-site field detection, and (iv) point-of-care testing [78]. For these reasons, the biosensor has been identified as a viable alternative method for the microbial screening, without any further treatment of the tested samples. 

In this respect, biosensors provided a diagnostic tool either for viruses or for neutralizing antibodies in many trials by obtaining signals, which, in the best scenario, were directly proportional to the viral load or concentration of the analytes [85,86,87]. To introduce the reader in biosensor-based diagnosis and monitoring of viral diseases, in this section we briefly review a few biosensing devices, which have been developed for previous viral outbreaks. Then we focus our attention on the small amount of readily developed devices useful in the present day outbreak. In doing that, we first focus on electrochemical devices, then on lateral-flow devices, and finally on colorimetric rapid tests. Essentially, electrochemically based biosensors operate through the detection of a specific biological analyte (protein, nucleotide, or metabolite) through its conversion into a proportional electrical signal for further analysis via transducers. In particular, electrochemical immunological techniques (EITs) could potentially replace ELISA and PCR for the monitoring of viral diseases. The EITs offer the advantage of the rapid and direct detection of the antigen–antibody interactions. Research directions towards the EIT developments are focused on finding new transducer materials, which are able to enhance immobilization along with the orientation of antibodies, increasing the sensitivity and dynamic range of detection. 

Accordingly, a MERS-CoV immunosensor was developed on an array of carbon electrodes modified with gold nanoparticles [88]. The electrodes were preliminary coated with recombinant MERS-CoV S protein, but also other hCoV antigens have been tested. The immunoassay relies on the competition of free (on the virus in the sample) and immobilized (on the electrode) MERS-CoV proteins in the presence of a known concentration of purified anti-S-protein antibodies that are added to the samples. The electrochemical response was monitored by using square wave voltammetry (SWV), exploiting ferrocyanide/ferricyanide as a redox probe, measuring the change in the electric current at a working potential of −0.05 V (versus Ag/AgCl). A remarkable linear response was investigated by using different concentrations of the MERS-CoV antigen, ranging from 0.001 to 100 ng/mL, and hCoV antigen, in the range of 0.01 to 10.000 ng/mL. The resulting LOD was 0.4 and 1 pg/mL for MERS-CoV and hCoV, respectively. Influenza A and B viruses were used in the selectivity testing, showing a high selectivity of assay towards MERS-CoV over these viruses. Besides, it provided a sensitive and easily performed technique for testing clinical samples, including the spiked nasal samples, within 20 min [88]. Though very attractive, as it virtually allows for whole virus detection and early diagnosis, such technology relies on high quantities of purified antibodies, which are generally available slightly later than outbreak onset, very often expensive, and sometimes difficult to produce on a large scale.

**Figure 9:**
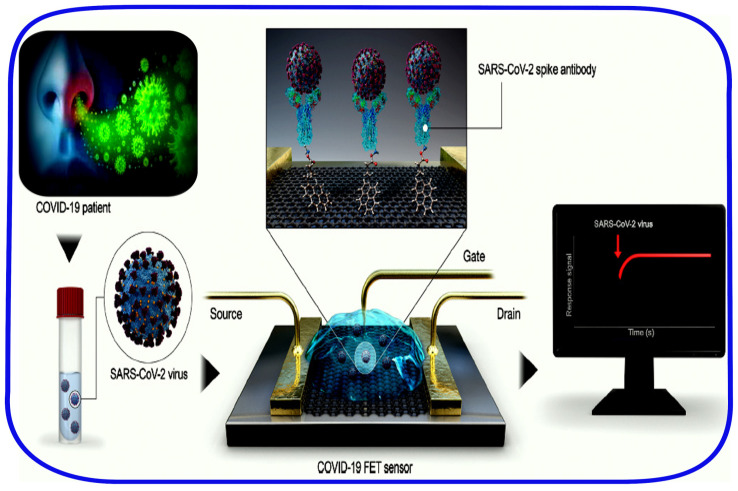
COVID-19 SARS-CoV-2 FET biosensor operation procedure. Graphene implemented as a sensing material, and SARS-CoV-2 spike antibody is conjugated onto the graphene sheet via 1-pyrenebutyric acid N-hydroxysuccinimide ester, which is an interfacing molecule as a probe linker. Figure adapted from [89] (Further permissions related to this figure should be directed to the ACS https://doi.org/10.1021/acsnano.0c02823).

Additionally, a sophisticated field-effect transistor (FET)-based biosensor (Figure 9) was designed to investigate the virus antigen in clinical samples, including the nasopharyngeal swabs and the cultured virus–cell suspension, without sample pretreatment or other further labeling [89]. Graphene sheets were functionalized by SARS-CoV-2 S-protein-specific antibodies to allow the SARS-CoV-2 detection in clinical samples. The FET was covered with a phosphate-buffered saline (PBS) buffer, pH 7.4, as the electrolyte to maintain an efficient gating effect to be used for the electrical signal transduction. The aqueous solution-gated FET system could detect SARS-CoV-2 based on changes in channel surface potential and the corresponding effects on the electrical response. The FET device was able to detect 1 fg/mL of SARS-CoV-2 spike protein in PBS with a LOD substantially lower than that of the ELISA platform. This value increases to a concentration of 100 fg/mL for the virus spike protein transported in 0.01x universal transport medium (UTM). Whereas, the FET device detects 242 copies/mL of spike protein in clinical samples of nasopharyngeal swabs diluted in UTM. Given that the UTM includes some reagents that may affect the sensor performance generating noise signals, the measured LOD could be low enough for practical use, considering that the detection limit of current molecular diagnostic tests for COVID-19 is ∼50–100 copies. In addition, the FET sensor clearly discriminated between patient and normal samples. Moreover, the device showed no remarkable cross-reactivity with commercial MERS-CoV spike proteins, indicating a high sensitivity and specificity for the SARS-CoV-2 spike antigen protein [89]. Nevertheless, novel materials development should be necessary to reduce the noise signals of UTM, for a more accurate detection.

**Figure 10:**
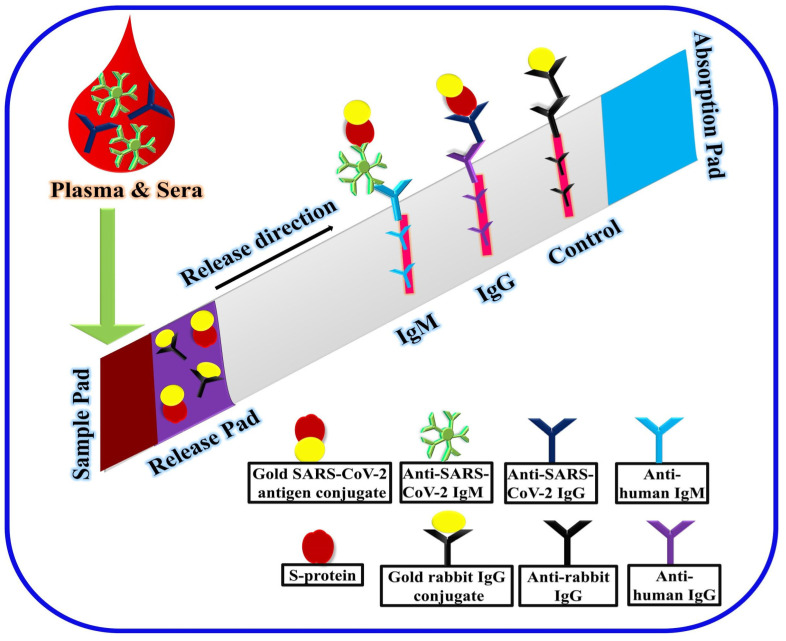
The point-of-care colloidal gold-immunochromatographic assay (GICA) for the rapid detection of SARS-CoV-2 antibodies.

As previously evidenced, though limited in terms of early diagnosis of an ongoing disease, immunological tests are still crucial in: (i) decentralizing rapid screening of potentially positive patients to relieve the eventually collapsing healthcare system (as experienced in Italy in March/April—running out of available beds and swabs); (ii) monitoring virus spread throughout the population—helping by taking countermeasures and more aware policies. Recently, a colloidal gold-immunochromatographic assay (GICA) was developed against SARS-CoV-2 by Li et al. [90]. Nevertheless, given that this assay is based on the GICA technology, it is very fast, easy to use, and easy to read. Such a rapid and simple point-of-care lateral flow immunoassay has been developed to detect human IgM and IgG antibodies against SARS-CoV-2 virus in blood. In fact, after the sample loading, the liquid is chromatographed upward under the capillary effect. Using immobilized antihuman-IgM, antihuman-IgG, and antirabbit-IgG (as a control) antibodies, a red line is formed when human IgM/IgG from the sample bind to immobilized anti-immunoglobulins (Figure 9). Color detection was carried out with a mixture of gold nanoparticles (AuNP) conjugated with recombinant S-protein antigen and of AuNP-rabbit-IgG antibody (Figure 10). The appearance of a single red line at the immobilized antihuman-IgM place points out the presence of only IgM in the analyzed sample, allowing the diagnosis of an early stage infection. The presence of two bands or a single red band at the antihuman-IgG place, indicates the presence in the analyzed sample of IgM/IgG or only IgG, respectively, allowing the diagnosis about the evolution of infection. In addition to its use as a diagnostic tool, the GICA test could also permit a low-cost monitoring of populations. In fact, this assay was used for the rapid screening of SARS-CoV-2 carriers, symptomatic or asymptomatic cases, and the observed sensitivity and specificity were 88.66% and 90.63%, respectively. Although the limit of the detection of the assay was not yet estimated, the result of this serological assay can be subsequently combined with the RT-PCR to improve the efficiency of the diagnosis [90]. A commercial GICA test (Zhuhai Livzon Diagnostics Inc., Zhuhai, China) was also developed. Interestingly, no significant differences of sensitivity and specificity between the ELISA and the GICA were observed (82.4% sensitivity, 100% selectivity) [75].

**Figure 11:**
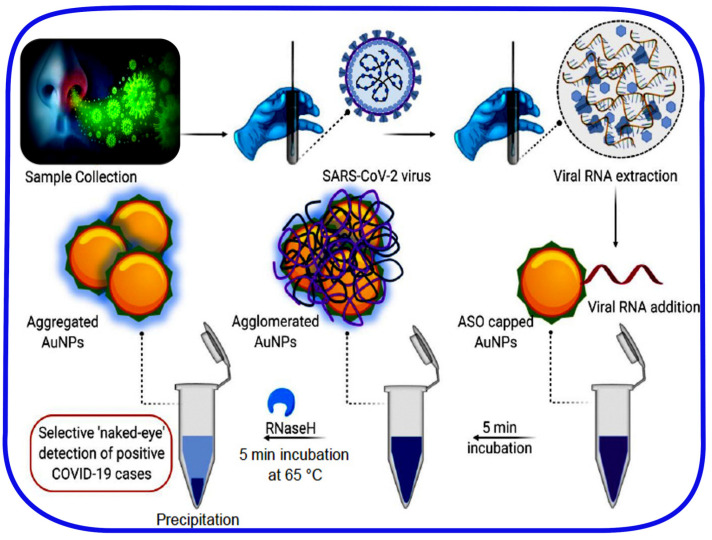
Mediation of SARS-CoV-2-RNA detection using suitably designed antisense oligonucleotides (ASOs)-capped gold nanoparticles (AuNPs). Figure adapted from [83]. (Further permissions related to this figure should be directed to the ACS https://doi.org/10.1021/acsnano.0c03822).

On the other hand, a direct colorimetric assay based on AuNPs capped with thiol modified antisense oligonucleotides (ASOs) was achieved. The used oligonucleotides were very specific for the nucleocapsid phosphoprotein (N protein) of SARS-CoV-2. This nanotechnology-based colorimetric bioassay for SARS-CoV-2-RNA detection requires preliminary viral RNA extraction and purification from the cellular lysate, carried out in few and easy steps using a commercially available kit. The color of the suspension changed from a violet to dark-blue color after the addition of SARS-CoV-2-extracted RNA, as a result of Au-ASO-RNA agglomerate formation. The addition of RNaseH cleaved the RNA strand from the RNA–DNA hybrid leading to a visible, detected precipitate mediated by the plasmonic signal response that was improved by the further deposition of more gold nanoparticles to the suspension. The selectivity of the technique was implemented in the presence of MERS-CoV-extracted RNA, and the estimated LOD was 0.18 ng/µL of the SARS-CoV-2 payload (Figure 11) [91]. The developed naked-eye instrument enabled a rapid detection of the targeted virus within 10 min from the extraction of the viral RNA from the positive infected patients with no need for advanced instrumentation. 

A viable alternative may be represented by virus imprinting technology (VIT), which involves the preparation of a synthetic polymeric matrix to capture the virus particles by molecularly imprinted polymers (MIP). The imprinting process is performed in the presence of the target as a whole virus particle, a single protein, or epitope to generate specific receptor/binding sites that have a high affinity for a targeted virus [92,93,94,95,96,97]. Such materials can be fabricated by different techniques (e.g., electropolymerization, microcontact printing, molding, and self-assembly), thus widening their applicability [98,99,100,101]. VIT has been used for the electrochemical detection of Zika virus using on-surface imprinted polymers and graphene oxide composites [102]. VIT was also used for the detection of human adenovirus (AdV), selected as a model virus facilitating the development and application of a rapid virus quantification [103]. Moreover, application of VIT resulted in the differentiation of different subtypes of human seasonal influenza (Influenza A virus) by integration of MIPs onto a quartz crystal microbalance (QCM) transducer. Development of virus-mediated MIPs integrated with QCM for the detection of the NS1 of Dengue virus in blood samples is achieved [92]. MIPs were fabricated based on fluorescent resonance energy transfer (FRET) for the sensitive diagnosis of Japanese encephalitis virus and the microcontact polymerization technique of dopamine in the presence of the epitope-gp41 for human immuno-deficiency virus (HIV) diagnosis to facilitate their early monitoring [93,104].

Recently, MIP-based SARS-CoV-2 detection has been suggested [105] and a preprint has appeared in which a MIP-based nanoparticle has been fabricated starting from the RBD of a SARS-CoV-2 S protein [106]. Such “monoclonal-type” plastic antibodies have been then tested only for in vitro rebinding of the RBD and hemocompatibility (for infusion in the patients). Nevertheless, such a nanodevice could be very promising for diagnostic application. Again, sufficient scrutiny should be preserved prior to the peer-review process.

## 3. Final Remarks and Future Outlook

The current COVID-19 crisis enforces the globe to immediately find opportune countermeasures in several fields. From the biological point of view, very little is still known about CoVs and how fast they can cross between species. Very little is known also about the mechanism of transfection to specific cells and tissues, as well as how long it takes to have a relevant immunological response and how long does the immunity, if any, persist. In particular, cellular tropism is highly desirable, especially how the RBD of the S1-domain selectively utilizes the host cell determinants (e.g., ACE2 for SARS-CoV and SARS-CoV-2; DPP4 for MERS-CoV) [40,83,84].

From the medical standpoint, infection and transmission of the virus is currently a very hot topic in the literature, and it is still not clear which are the physiological and genetic reasons why few people do not show any evident symptomatology. Moreover, there is still some debate about the best treatments for the very aggressive pneumonia that affected and is still affecting a great number of people [107]. A general cure for different CoVs is another urgent need, both in terms of prevention by vaccination and in terms of treatment (e.g., retroviral drugs, neutralizing plasma, purified antibodies, inhibitors). 

In this review, we mainly focused on the diagnostic and monitoring techniques. Reliable, secure, noninvasive, and affordable methods are necessary more than ever during a pandemic, to quickly report the positive cases and reduce the numbers of contagions/infections. We first discussed techniques that mostly rely on cell cultivation (Table 1). Though very useful for clinical study, such techniques suffer a high level of risk (BSL3) that the operators are subjected to. Even when alternatives can be engineered in which native antigens are implanted into safer viruses, very long procedures must be performed. 

Given this, the actual gold standard is represented by the genetic analysis through RT-PCR (Table 1). In the present SARS-CoV-2 outbreak, nasopharyngeal and/or oropharyngeal swabs, and successive cloning, amplification, and sequencing have been widely adopted. However, this approach needs at least three hours and specialized reagents, which have sometimes been difficult to find when the number of patients per day increases exponentially. Nevertheless, the nature of the techniques also give the opportunity to rapidly monitor for virus mutations, which may be extremely helpful in adapting the right countermeasures. CRISPR-Cas12 cassette is increasingly emerging as a breakthrough technique, which holds the promise to revolutionize genetic sciences (Table 1). In this regard, an extremely fast method has been previously described, even though we fear that it would be difficult to spread given that such technology is somehow still a niche. 

In a different way, immunoassays and antigen detection methods may play an outstanding role in the containment of the disease. Their power in early diagnosis is limited, as it has been shown that specific antibodies could be revealed only after one week from the disease onset by the commercially available serological tests; the latter are extremely useful in monitoring population on a wider basis and find useful sociological and geographic correlations. 

A collaborative effort (COVID-19 MS Coalition) has been tackled with the aim of sharing MS research data in order to promote the development of fast and sensitive tests for both COVID-19 diagnosis and monitoring. In fact, MS-based detection of specific peptide sequences that derive from SARS-CoV-2 proteins is now under development, as well as useful biomarkers for recognizing the course of the disease. Moreover, very fast chromatographic analysis, together with MS/MS determination, should now enable expert users to determine virulence with very high precision. However, the high cost of instruments, together with the necessary high skill of personnel carrying out the analysis, makes its possible application less amenable from a diagnostic point of view.

In this context, the global dilemma of the hCoV outbreaks has encouraged researchers to exploit every moment to develop and fabricate on-site, point-of-care, and easily portable and affordable tests. Ideally, they should be easily carried out and should not require any sample pretreatment. Such devices would represent a breakthrough to be used in hospitals and airports. The use of biosensors would represent one of the best choices against the actual emergency (as well as future threats), allowing cheap and noninvasive monitoring systems, with fast response (<1 min) and, above all, a low background noise. Nowadays, biosensors are already of common use, such as electrochemistry-based glucometers for glucose determination in blood [108,109] or lateral-flow-based devices for fertility and pregnancy tests [110,111]. Other biosensors have been proposed for several applications in healthcare, such as wearable biosensors for healthcare monitoring [112] for the detection of toxic substances in human fluids [113] or in foods [114], including possible real-time detection in the environment [115,116,117,118]. The simpler its use, the more a biosensor can be introduced into daily life with user-friendly self-explanatory interfaces. Therefore, the development of appropriate biosensors will have effects at different levels for the containment of the virus spreading through a fast and wide monitoring of people. 

Nevertheless, these methods inspire scientists to develop quantitative, rapid, and affordable assays for the hCoV detection to cope with the current situation and to reduce the burden of the expenses required by the use of more sophisticated tools. In this review, we covered some of the SARS-CoV-2-specific biosensors that have been published so far, but the field is rapidly growing, and many more have just been published during the writing of this manuscript [119]. Electrochemical immunosensors, as the FET-based on previously discussed, hold the promise of being a cheap and reliable solution in the direct recognition of the virus in real case scenarios. In this respect, also antisense oligonucleotides-capped gold nanoparticles could be extremely useful, as such eye-based technologies have the advantage of being fast and rapidly applicable in the field during the emergency of a virus outbreak. Very recently, based on a similar approach, a patent by a Neapolitan spin-off is pending on rapid detection (3 min) of SARS-CoV-2 by the common effort of industry and university (http://www.cosvitec.com/index.php/it/). Nevertheless, both technologies rely on nasopharyngeal or oropharyngeal swabs that cannot be self-performed and that could eventually damage oral mucosa. Operators should wear suitable personal protective equipment prior to virus inactivation in the storage solution anyway. These reasons represent a great burden to the spread of such technologies as point-of-care (PoC) devices in virus detection and disease diagnosis. In perspective, such biosensors could be adapted and installed to “breath-sampling” touch-free devices (e.g., spirometers, balloons, face masks). As an alternative, serological sampling is also within the ability of the unskilled end user. As previously reported, the IgM/IgG immunoassay (GICA) can screen the patients for disease in a few minutes (Table 1) by using a blood drop. As previously described, this assay does not detect the virus in the early stage of disease. Therefore, a weekly test would be needed in this respect to help in outbreak monitoring and control, helped by the PoC nature of such devices. However, this method is not a quantitative assay, and the limit of detection is not yet estimated, making the diagnostic confirmation by other techniques still necessary.

In this scenario, there is still room for improvement, and ample work must be performed in the development of a self-consistent PoC device that is capable of not only detecting the virus but also checking for disease progression. Interesting advancements may eventually be provided by MIPs. Such polymeric matrices (tailor-made plastic antibodies) can be pursued and optimized for SARS-CoV-2 detection due to their robustness, sensitivity, and selectivity through the creation of the specific recognition cavities, in addition to cost-efficiency and their long-term stability. Also, further advancement in this class of detection methods may be provided by de novo protein design [120,121,122,123,124]. De novo-designed proteins are totally unrelated to any natural sequences. Such proteins generally show reduced size, high levels of expression, and high stability. In a recent publication, Sesterhenn et al. [125] designed de novo proteins around well-known antigen peptides with the aim to stabilize their structure. It has been shown that such small de novo proteins are able to elicit an immune response, inducing physiologically relevant neutralizing serum levels [125]. This revolutionary approach not only may be helpful in creating epitope-specific vaccines, but it may constitute a viable alternative to the recombinant expression of viral proteins and/or synthesis of peptide libraries for diagnostic purposes (e.g., ELISA, GICA, magnetic beads). Additional improvement may come from artificial reporter enzymes [124,126,127,128]. These miniaturized metalloenzymes are able to drastically increase the active site density, by conjugating a higher number of them either to antibodies or to gold nanoparticles, compared with generally adopted reporter enzymes (e.g., HRP, luciferase).

## Figures and Tables

**Table 1:** Monitoring and detection methodologies for SARS-CoV-2.

Detection	Target Analyte	Source	Time	LOD	Sensitivity	Selectivity	On Market	Ref.
***Cell culture***								
CPE ^1^	Virus	Swabs ^4^	2–3 days	-	-	-	No	63
MNA ^2^	Human IgM and IgG	Plasma/Serum	2–4 days	10,000 TCID_50_/mL	-	100%	No	63
***Genetic***								
RT-PCR	Viral RNA	Swabs ^4^	3–4 h	1 copy/μL of input ^7^	95%	100%-	Yes	64
CRISPR-Cas12 assay	E gene and N gene	Swabs ^4^	30–45 min	10 copies/μL of input	95%	-	No	67
***Immunological***								
ELISA	Human IgM and IgG	Plasma/Serum	3–4 h	-	44.4% ^8^ 82.5% ^9^	100%	Yes	76
Chemiluminescent Immunoassay	Human IgM and IgG in a separate assays	Serum	48 min	-	100% ^9^	57.2% ^8^ 71.4% ^9^	Yes	78
Proteomic *								
LC-MS	Virus proteins	Swabs ^4^	1–2 h	10,000 particles	-	-	No	82
***Biosensig device***								
FET-based biosensor	SARS-CoV-2 S-protein	Swabs ^5^ Cultured virus-cell suspension	3–10 min ^6^	242 copies/mL	-	100%	No	90
GICA	Human IgM and IgG	Plasma/Serum	15 min	-	88.66%	90.63%	Yes	91
ASOs-Capped Plasmonic Nanoparticles ^3^	SARS-CoV-2 N-protein	Swabs ^4^	~1 h	0.18 ng/µL	-	100%	No	92

^1^ Cytopathogenic effects; ^2^ Microneutralization assay; ^3^ Naked-Eye Detection: ^4^ Nasopharyngeal and oropharyngeal; ^5^ Nasopharyngeal; ^6^ Measuring time from the virus payload in the sample; ^7^ Limit of detection of RT-PCR Diagnostic Panel (QIAGEN QIAmp DSP Viral RNA Mini Kit): ^8^ for IgM; ^9^ for IgG. * These data are not confirmed, because are from pre-prints not peer-reviewed.
